# Supplementary material for: STRONG: metagenomics strain resolution on assembly graphs
Source: Genome Biol. 2021 Jul 26;22:214. doi: 10.1186/s13059-021-02419-7 (PMC8311964; doi:10.1186/s13059-021-02419-7)
Supplement: Supplementary file 1 — Additional file 1 Supplementary tables and figures. [file 13059_2021_2419_MOESM1_ESM.pdf]

Supporting information

Supplementary tables

Species name	Tax. ID.	Strain ID	No. contigs	Genbank Accn no.
Pseudomonas chlororaphis	587753	1037915	1	GCF˙000264555.1
Pseudomonas chlororaphis	587753	86192	1	GCF˙000761195.1
Pseudomonas chlororaphis	587753	587753	1	GCF˙000698865.1
Pseudomonas chlororaphis	587753	1038921	1	GCF˙000281915.1
Bordetella holmesii	35814	1247648	4	GCF˙000598125.1
Bordetella holmesii	35814	1172205	1	GCF˙000765395.1
Bordetella holmesii	35814	1247646	4	GCF˙000572015.1
Bordetella holmesii	35814	1266729	2	GCF˙000341465.1
Comamonas testosteroni	285	399795	1	GCF˙000168855.1
Comamonas testosteroni	285	1392005	1	GCF˙000739375.1
Acetobacter pasteurianus	438	438	1	GCF˙001183745.1
Rhodococcus erythropolis	1833	234621	4	GCF˙000010105.1
Rhodococcus erythropolis	1833	1136179	2	GCF˙000454045.1
Rhodococcus erythropolis	1833	1289591	3	GCF˙000696675.1
Rickettsia prowazekii	782	272947	1	GCF˙000195735.1
Clostridium sporogenes	1509	1509	1	GCF˙001020205.1
Clostridium sporogenes	1509	471871	2	GCF˙000155085.1
Mycoplasma capricolum	2095	1124992	1	GCF˙000835085.1
Mycoplasma capricolum	2095	40480	1	GCF˙000953375.1
Achromobacter xylosoxidans	85698	1167634	1	GCF˙000967095.2
Achromobacter xylosoxidans	85698	562971	1	GCF˙000758265.1
Sinorhizobium meliloti	382	1230587	4	GCF˙000304415.1
Sinorhizobium meliloti	382	698936	3	GCF˙000147775.2
Mycoplasma gallisepticum	2096	1159201	1	GCF˙000286755.1
Mycoplasma gallisepticum	2096	710128	1	GCF˙000025365.1
Mycoplasma gallisepticum	2096	708616	1	GCF˙000025385.1
Riemerella anatipestifer	34085	1228997	1	GCF˙000295655.1
Riemerella anatipestifer	34085	1271752	1	GCF˙000331695.1
Riemerella anatipestifer	34085	693978	1	GCF˙000183155.1
Riemerella anatipestifer	34085	1455062	1	GCF˙001077795.1
Riemerella anatipestifer	34085	992406	1	GCF˙000191565.1
Leuconostoc mesenteroides	1245	33966	2	GCF˙001047695.1
Bacillus atrophaeus	1452	720555	1	GCF˙000165925.1
Bacillus atrophaeus	1452	1239783	1	GCF˙000385965.2
Lactobacillus fermentum	1613	1381124	2	GCF˙000466785.3
Anaplasma phagocytophilum	948	1184254	1	GCF˙000439795.1
Borreliella garinii	29519	1081646	3	GCF˙000239475.1
Borreliella garinii	29519	1421551	1	GCF˙000691545.1
Borreliella garinii	29519	1234596	1	GCF˙000300045.1
Flavobacterium psychrophilum	96345	96345	1	GCF˙000739395.1
Flavobacterium psychrophilum	96345	96345	1	GCF˙000971645.1
Flavobacterium psychrophilum	96345	1452724	1	GCF˙000754405.1
Coxiella burnetii	777	227377	2	GCF˙000007765.1
Coxiella burnetii	777	434924	2	GCF˙000019885.1
Coxiella burnetii	777	360115	2	GCF˙000018745.1
Bradyrhizobium japonicum	375	375	1	GCF˙000807315.1
Salinispora tropica	168695	369723	1	GCF˙000016425.1
Brucella ovis	236	444178	2	GCF˙000016845.1
Agrobacterium tumefaciens	358	1435057	4	GCF˙000576515.1
Neorhizobium galegae	399	1028801	3	GCF˙000731295.1

Table S1: **Genomes used in the synthetic communities (part I).**

Species name	Tax. ID.	Strain ID	No. contigs	Genbank Accn no.
Lactobacillus jensenii	109790	575606	4	GCF˙000161895.2
Corynebacterium glutamicum	1718	1079988	2	GCF˙000233355.2
Haloferax mediterranei	2252	523841	4	GCF˙000306765.2
Haloferax mediterranei	2252	523841	4	GCF˙000685635.1
Haemophilus parainfluenzae	729	862965	1	GCF˙000210895.1
Streptococcus dysgalactiae	1334	617121	1	GCF˙000307185.1
Lactobacillus salivarius	1624	712961	4	GCF˙000143435.1
Yersinia frederiksenii	29484	1454377	2	GCF˙000834215.1
Edwardsiella tarda	636	498217	2	GCF˙000020865.1
Bacillus pumilus	1408	315750	1	GCF˙000017885.1
Bacillus licheniformis	1402	1402	1	GCF˙000876525.1
Bacillus licheniformis	1402	1402	4	GCF˙000948275.1
Saccharolobus solfataricus	2287	2287	1	GCF˙000968435.1
Morganella morganii	582	1124991	1	GCF˙000286435.2
Ralstonia solanacearum	305	859656	1	GCF˙000197855.1
Ralstonia solanacearum	305	1031711	2	GCF˙000215325.1
Ralstonia solanacearum	305	305	1	GCF˙001373335.1
Ralstonia solanacearum	305	305	2	GCF˙001299555.1
Ralstonia solanacearum	305	305	1	GCF˙001373255.1
Pantoea ananatis	553	706191	1	GCF˙000025405.2
Pantoea ananatis	553	932677	2	GCF˙000270125.1
Pantoea ananatis	553	1123863	2	GCF˙000283875.1
Pantoea ananatis	553	1095774	2	GCF˙000233595.1
Bartonella bacilliformis	774	1293904	4	GCF˙000709855.1
Bartonella bacilliformis	774	1293906	3	GCF˙000709775.1
Bartonella bacilliformis	774	1293907	4	GCF˙000709875.1
Bartonella bacilliformis	774	1293910	4	GCF˙000709755.1
Actinobacillus pleuropneumoniae	715	537457	4	GCF˙000020405.1
Actinobacillus pleuropneumoniae	715	416269	1	GCF˙000015885.1
Sulfolobus islandicus	43080	429572	1	GCF˙000022385.1
Sulfolobus islandicus	43080	1132501	1	GCF˙000245175.1
Sulfolobus islandicus	43080	427317	1	GCF˙000022405.1
Sulfolobus islandicus	43080	930943	1	GCF˙000189575.1
Streptococcus thermophilus	1308	1308	1	GCF˙000971665.1
Streptococcus thermophilus	1308	264199	1	GCF˙000011825.1
Streptococcus thermophilus	1308	1187956	1	GCF˙000262675.1
Streptococcus thermophilus	1308	1308	1	GCF˙001008015.1
Streptococcus thermophilus	1308	767463	1	GCF˙000182875.1
Methanosarcina barkeri	2208	1434109	2	GCF˙000969985.1
Methanosarcina barkeri	2208	796385	1	GCF˙001027005.1
Methanosarcina mazei	2209	1434114	1	GCF˙000970225.1
Methanosarcina mazei	2209	1434115	1	GCF˙000970185.1
Methanosarcina mazei	2209	213585	1	GCF˙000970205.1
Methanosarcina mazei	2209	1434117	1	GCF˙000970165.1
Methanosarcina mazei	2209	192952	1	GCF˙000007065.1
Bifidobacterium bifidum	1681	702459	1	GCF˙000165905.1
Bifidobacterium bifidum	1681	883062	1	GCF˙000164965.1
Bifidobacterium bifidum	1681	1681	2	GCF˙001020375.1
Bifidobacterium bifidum	1681	500634	1	GCF˙001025135.1
Bifidobacterium bifidum	1681	398513	1	GCF˙000273525.1

Table S2: **Genomes used in the synthetic communities (part II).**

Data set	Synth_03	Synth_05	Synth_10	Synth_15	AD
Size (Gbp)	45	45	45	45	116.3
No. of samples	3	5	10	15	10
No. of MAGs	34	37	39	40	304
Assembly	16.92	15.63	14.50	9.98	49.93
Binning	0.03	0.90	0.60	0.03	77.30
Subgraphs	0.60	1.58	0.90	0.93	35.05
BayesPaths	27.43	35.1	36.82	19.4	228.65

Table S3: **Approximate STRONG run times in hours for data sets in this study.** We give time in hours for the major steps of the STRONG pipeline. These were run using 64 cores of a 192 core Linux x86_64 server running Intel(R) Xeon(R) CPUs E7-8850 v2 @ 2.30GHz.

Method	Data set	MAGs	$\langle CV \rangle$	#SCGs	#fSCGs	Found	Not F.	Rep.	Err	R^2	f^G
STRONG	Synth_T1_S10	8	1.45	31.875	25.375	27	8	—	0.00078	0.99 (0.99)	1/8 = 0.125
STRONG	Synth_T10_S10	10	0.65	31.1	24.1	35	10	2	0.086	0.95 (0.99)	4/10 = 0.4
STRONG	Synth_T100_S10	8	0.23	32.5	27.625	27	8	4	0.38	0.97 (0.99)	3/8 = 0.375

Table S4: **Impact of strain variability on STRONG reconstruction accuracy.** Results are shown for the three data sets with increasing levels of precision in strain proportions Synth_T1_S10, Synth_T10_S10 and Synth_T100_S10. MAGs: The number of MAGs found. $\langle CV \rangle$: average strain coefficient of variation *i.e.* standard deviation of relative abundance divided by mean. #SCGs: The average number of SCGs found in each MAG. #fSCGs The average number of SCGs after filtering in STRONG. Found: Number of reference strains that had a predicted strain that best matched it. Not F.: Number of reference strains that had no predicted strain with a closest match to it. Rep.: Number of strains matching to a reference that already has a better match. Err: The average error rate of the 'Found' strains in percentage base pairs. R^2: Correlation between predicted and actual strain relative proportions given as adjusted R^2, the figure in parentheses is when restricted to MAGs where the number of strains was correctly predicted. f^G: the fraction of MAGs where the number of strains was correctly inferred.

Sample	Week	Nucleotides	Temperature	H2S	CH4	O2
AD7˙W5˙Repeat2	5	1.48e+10	38.6	71.6	55.6	0.616
AD7˙W10	10	1.34e+10	36.8	267	52.9	0.299
AD7˙W14	14	1.15e+10	39	111	63.3	0.499
AD7˙W20	20	1.02e+10	39.1	329	57.3	0.299
AD7˙W25	25	1.26e+10	39.4	191	56.6	0.25
AD7˙W27	27	1.04e+10	39.5	423	56.3	0.467
AD7˙W30	30	9.57e+09	39.7	268	58.3	0.71
AD7˙W35	35	1.52e+10	39.9	233	55.4	0.703
AD7˙W40	40	9.54e+09	39.8	146	54.4	0.641
AD7˙W45	45	9.09e+09	39.9	23.4	55.7	0.419

Table S5: **Anaerobic digester time series samples.**

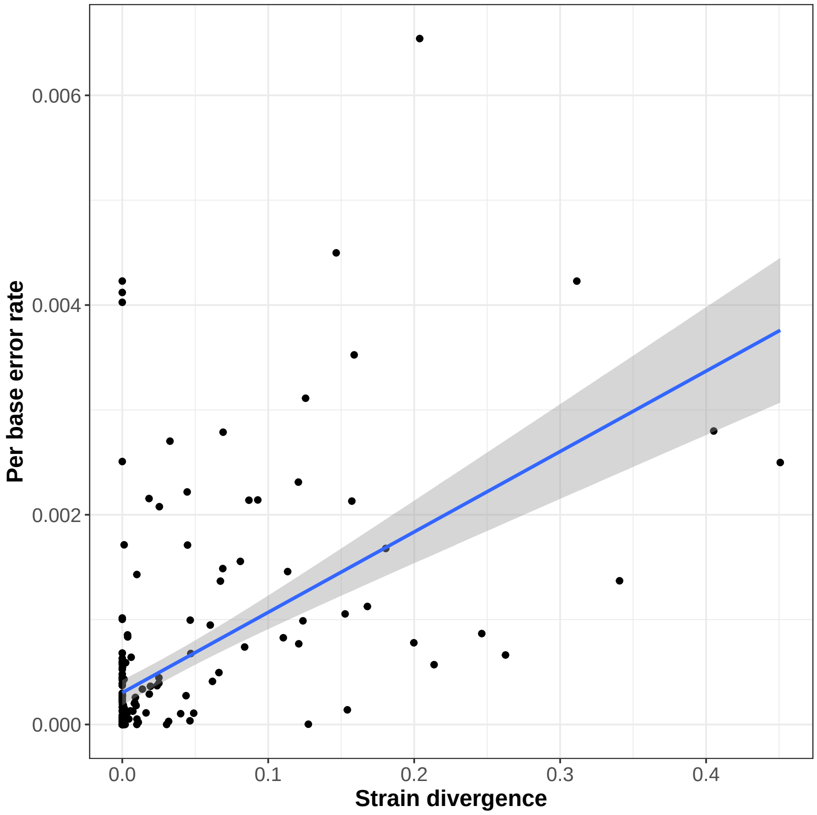

Figure S1: **Correlation between strain divergence and error rate for the synthetic community data.** For the four synthetic community data sets combined, Synth_03, Synth_05, Synth_10 and Synth_15, we show the estimated error as path divergence (see main text) against actual nucleotide error rates. The straight line is a linear regression. The error rates and divergences were correlated (Pearson's correlation: $r = 0.54$, $p < 2.2e - 16$).

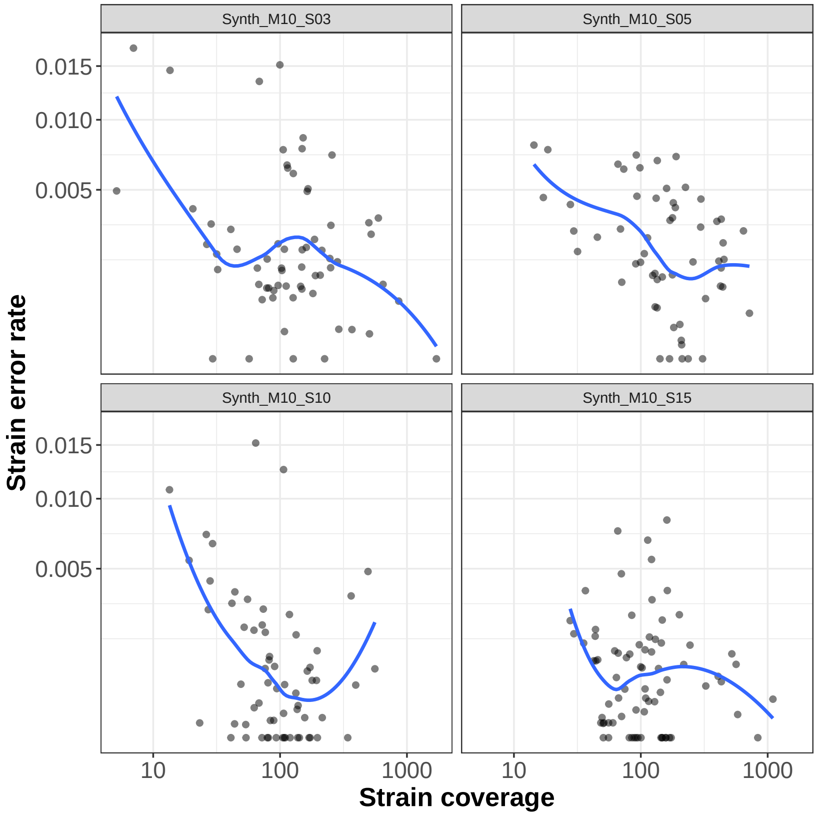

Figure S2: **Error rates in 'Found' strains against coverage depth for STRONG in the high diversity synthetic community data sets.** For the 'Found' strains we computed per base error rate to the matched reference, this is shown on the y-axis, against strain total coverage depth summed across samples on the x-axis, both axes are log transformed. The results are separated across sample number in the synthetic community.

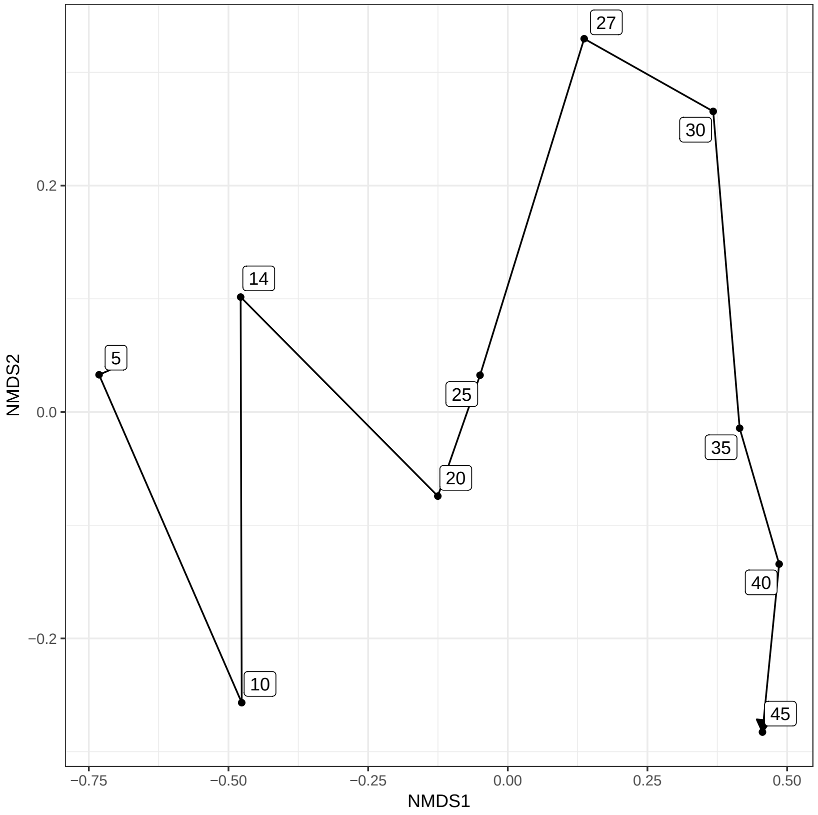

Figure S3: **NMDS plot of reactor community structure.** Bin coverages were normalised by per sample sequencing depth and Bray-Curtis distances calculated prior to NMDS, labels indicate sampling week. The sampling week had the strongest association with community structure ($R^2 = 0.46$, $p = 0.001$) followed by H_2S concentration ($R^2 = 0.17$, $p = 0.009$) and O_2 ($R^2 = 0.14$, $p = 0.014$) based on PERMANOVA with Bray-Curtis distances (Adonis function in vegan library R). The other operating conditions, temperature and CH_4, were not significantly associated.

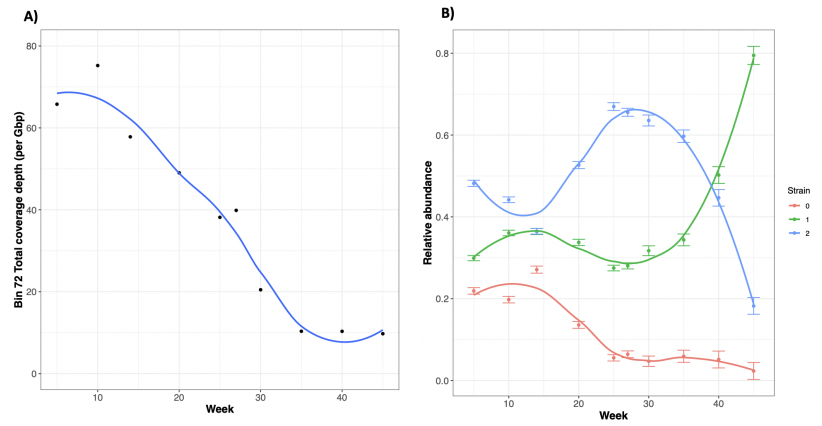

Figure S4: **Time series of A) total MAG coverage depth and B) STRONG strain abundances from Bin_72 of the AD time series**. A) Coverage depth of Bin_72 normalised by Gbp of sequence in each sample. This MAG decreased significantly in abundance over time (Pearson's correlation on log coverage $r = -7.9$ Benjamini-Hochberg adjusted $p = 4.9e - 05$). B) Strain relative proportions as calculated by BayesPaths with uncertainties as twice the standard deviation in the variational Bayesian prediction. Curves are LOESS smoothings of data points. Strain proportions did not change significantly (permutation multivariate ANOVA $R^2 = 0.35$ adjusted $p = 0.089$).

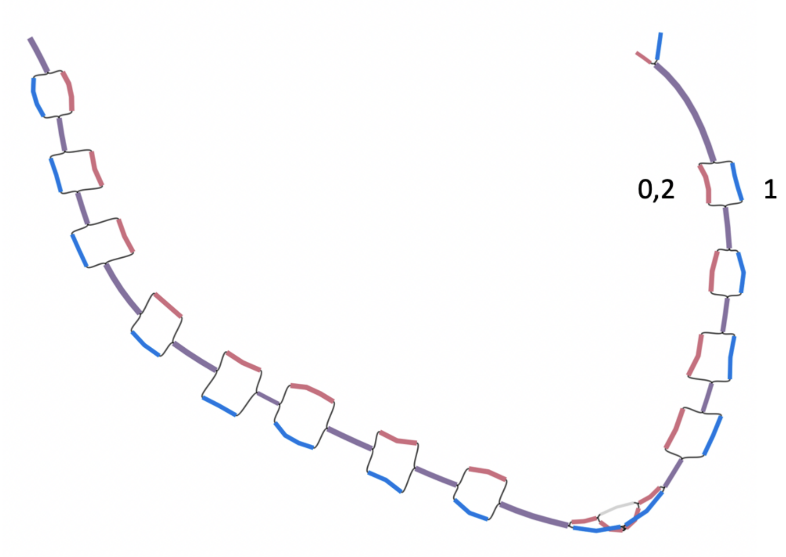

Figure S5: **Simplified variant graph for COG0532 from Bin_72 of the AD time series**. This is a bandage plot of the high-resolution subgraph extracted for COG0532 post-simplification. The colours indicate which unitig is present in which strain (0,1 and 2). In this case strains 0 and 2 are identical for this gene.

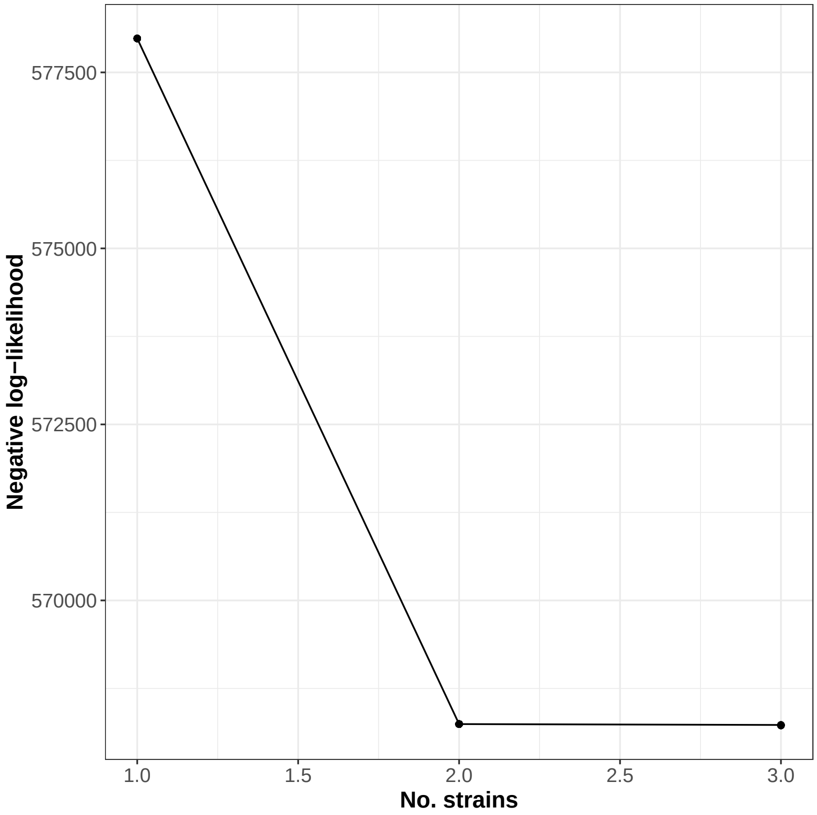

Figure S6: **Negative log-likelihood of Nanopore haplotype fits as a function of strain number for COG0532 from Bin_72 of the AD time series data set.** The hybrid EM algorithm for Nanopore strain resolution defined in the Methods was applied to all 1,603 Nanopore reads mapping to this SCG and the negative log-likelihood computed for ten replicates at each strain number. The algorithm was run for up to 4 strains but degenerate haplotypes are collapsed and in practice no more than three strains were ever observed in this case.

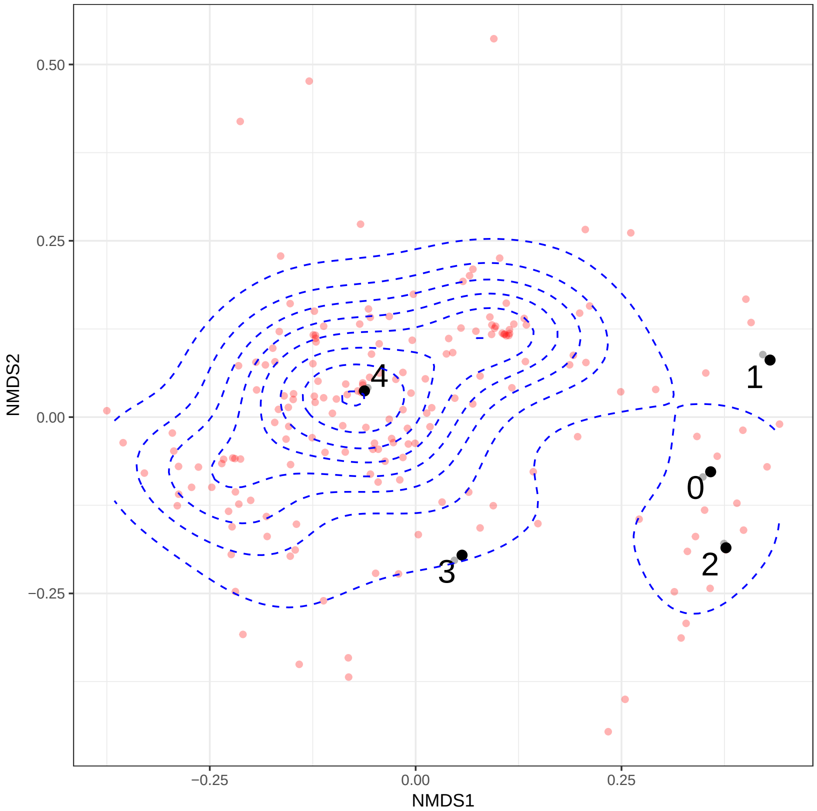

Figure S7: **Comparison of Nanopore reads to STRONG prediction for COG0072 from Bin_846.** Non-metric multidimensional scaling of Nanopore reads that mapped to COG0072 from Bin_846 of the anaerobic digester time series (red) together with the five haplotypes reconstructed from short reads by STRONG (black 0, 1, 2, 3 and 4). Distances were calculated as fractional Hamming distances on short read variant positions (see Methods). Blue dashed lines indicate read density contours. In this sample (Week 27) the short read haplotypes were predicted to have relative abundances $\rho_0 = 0.043 \pm 0.012$, $\rho_1 = 0.024 \pm 0.014$, $\rho_2 = 0.13 \pm 0.017$, $\rho_3 = 0.059 \pm 0.018$ and $\rho_4 = 0.74 \pm 0.027$.

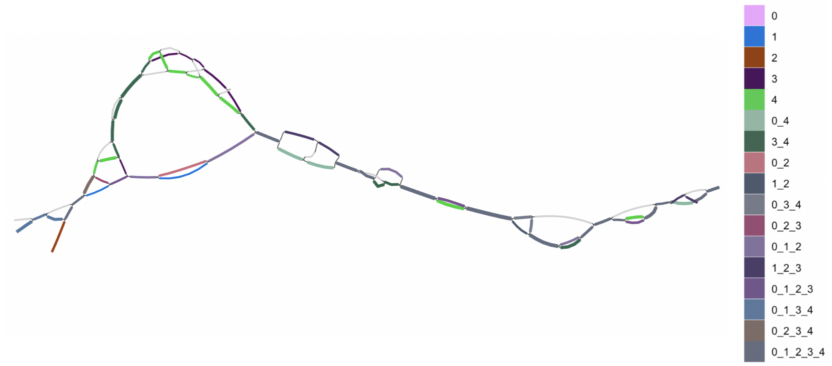

Figure S8: **Simplified variant graph for COG0072 from Bin_846 of the AD time series**. This is a bandage plot of the high-resolution subgraph extracted for COG0072 post-simplification. The colours indicate which unitig is present in which strain (see legend).

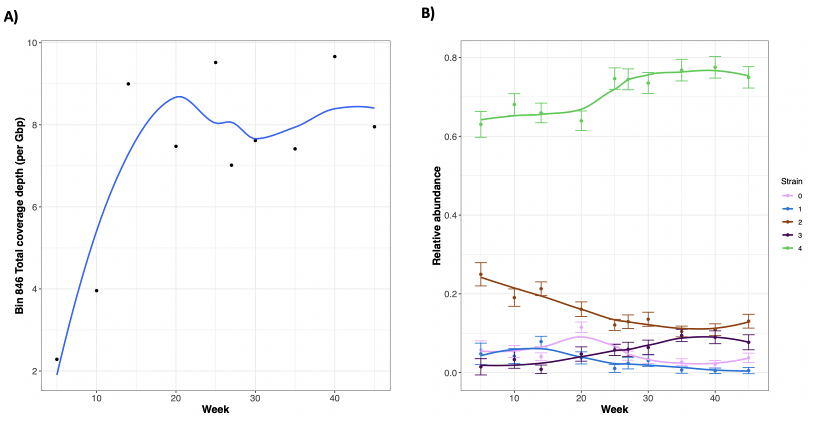

Figure S9: **Time series of A) total MAG coverage depth and B) STRONG strain abundances from Bin_846 of the AD time series**. A) Coverage depth of Bin_846 normalised by Gbp of sequence in each sample. This MAG increased marginally in abundance over time (Pearson's correlation on log coverage $r = 2.6$ Benjamini-Hochberg adjusted $p = 0.071$). B) Strain relative proportions as calculated by BayesPaths with uncertainties as twice the standard deviation in the variational Bayesian prediction. Curves are LOESS smoothings of data points. Strain proportions did change significantly (permutation multivariate ANOVA $R^2 = 0.72$ adjusted $p = 0.011$).

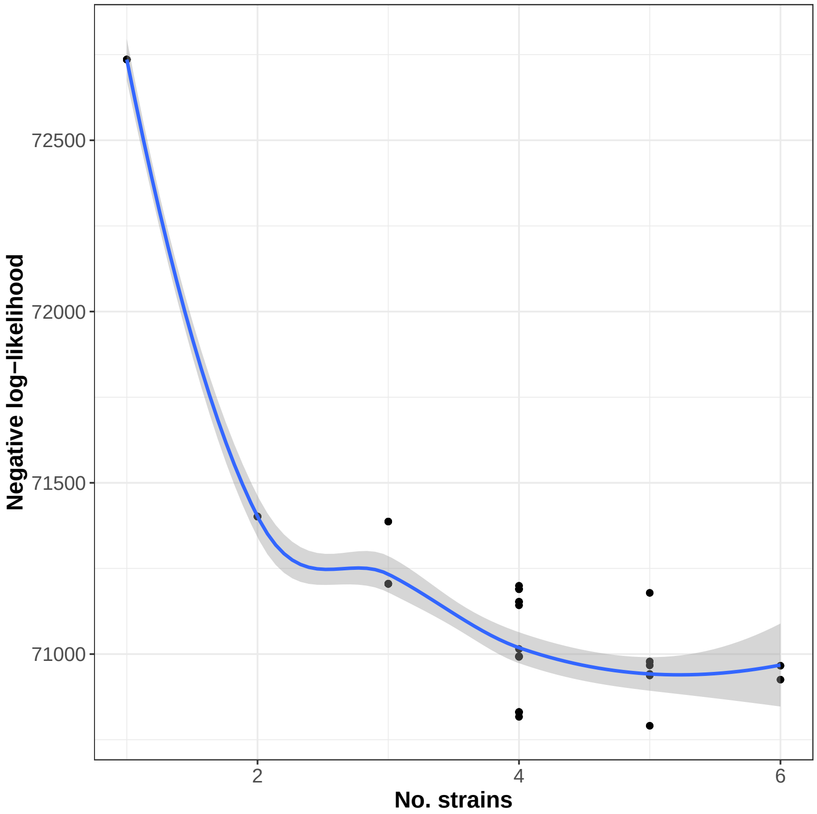

Figure S10: **Negative log-likelihood of Nanopore haplotype fits as a function of strain number for COG0072 from Bin_846 of the AD time series.** The hybrid EM algorithm for Nanopore strain resolution defined in the Methods was applied to the 194 Nanopore reads mapping to this SCG and the negative log-likelihood computed for ten replicates at each strain number. The algorithm was run for up to 6 strains.

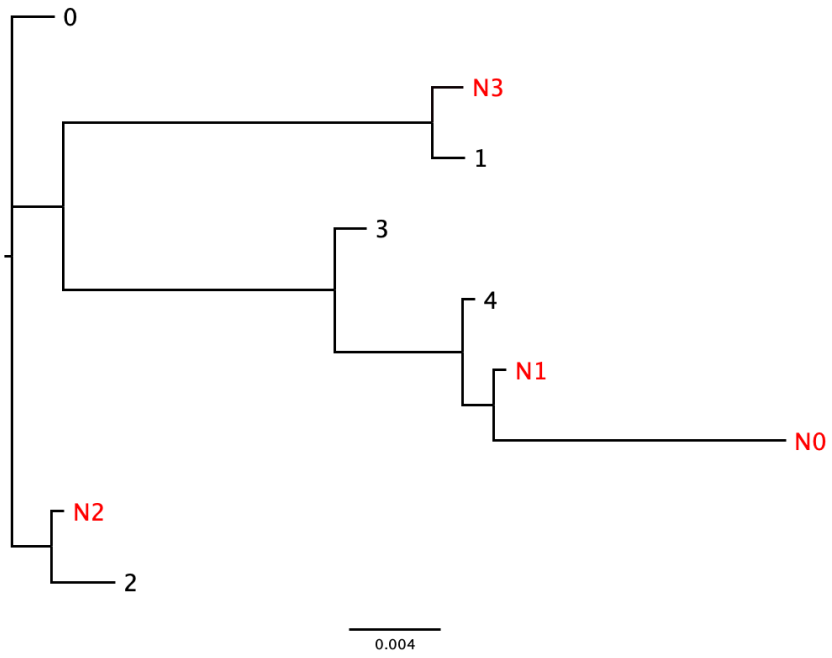

Figure S11: **Comparison of Nanopore and STRONG strain haplotypes for COG0072 from Bin_846 of the AD time series**. The four Nanopore haplotypes from the optimum run of the EM algorithm are shown in red and 5 STRONG haplotypes in black. The Nanopore haplotypes had relative abundance $\rho_0 = 0.056$, $\rho_1 = 0.820$, $\rho_2 = 0.091$ and $\rho_3 = 0.035$. N0 matched best to 4 with 98.8% nucleotide identity, N1 to 4 with 99.9%, N2 to 0 with 99.7%, N3 to 1 with 99.8%.
