# Supplementary material for: STRONG: metagenomics strain resolution on assembly graphs
Source: Genome Biol. 2021 Jul 26;22:214. doi: 10.1186/s13059-021-02419-7 (PMC8311964; doi:10.1186/s13059-021-02419-7)
Supplement: Supplementary file 3 — Additional file 3 Review history. [file 13059_2021_2419_MOESM3_ESM.docx]

**1^st^ round**

**Reviewer 1**

This paper introduces new software called STRONG, which enables strain resolved metagenomics using uncollapsed assembly graphs. The question of how to extract strain information present in metagenomes is an important research area that is at the leading edge of microbiome studies; STRONG potentially represents a substantial advance in research capabilities.

The underlying idea is clear, if not easy to implement: STRONG looks at the abundances of paths in cDBG unitigs across samples, and "resolves" paths through single copy gene sequences that best match the abundances, thus deconcolving them to match to different strains.

The paper is well organized, and the introduction and discussion are expert and excellent. The methods as presented also are clear and high quality. However, we struggled to understand exactly what the output of STRONG was, and how it could be used. Some specific questions here:

* what information does STRONG require to do its work? It seems like good quality genome assemblies/MAGs could be critical inputs, but we couldn't tell for sure.

* If good bins are required, are there concerns about providing this input, in terms of STRONG's output quality?

* what is the actual primary output of STRONG? the strain by sample abundance matrix? Is anything done with non-single-copy-gene sequences?

* does STRONG provide insight into accessory/non-core elements of strains?

* is STRONG limited to bacteria and archaea by the single-copy-gene paradigm used?

* does STRONG work only on time series, or it can be used on spatial series (at least in theory)?

* It seems like STRONG deals specifically with coding sequences (p20, line 8) in normal codon encodings. If so, that approach/limitation should be mentioned early on, as it limits STRONG to coding regions etc.

Other major issues/questions --

It seems like having STRONG built on top of DESMAN (STRONG postprocesses DESMAN output, we think?) would affect the comparison to DESMAN - that is, it's good it improves upon DESMAN, but would that not be expected? Are there other tools to compare to in terms of outputs? What kind of ground truth can be used to validate STRONG, if any?

Figure 2 was somewhat confusing in its presentation at first. If there is a way to use layout constraints or define the layout seed to keep haplo.0, haplo.1, and haplo.2 in the same layout but with different colors, that would make the figure clearer.

It is unclear what information Figure 6 contributes specifically.

We had a number of suggestions but aren't sure what the main message is, so please feel free to disregard these specific suggestions -

* perhaps limit the diagram to major phyla? Firmmicutes, Bacteroides, etc?

* We're not sure what the various FALSE etc. colors represent and they are impossible to distinguish on the figure.

p15, line 48, we're not sure what "complete metagenome de novo strain resolution will become a realistic possibility" means in terms of the approach presented here. What would be the specific outcome - completely strain-resolved MAGs with haplotype linkages? Please define!

The acknowledgements mention a contribution that seems significant enough for authorship.

Minor comments --

Paragraph beginning at line 49, p2 - it seems like strain variation belongs in here as a third reason? Also, after citation [17], a new sentence could be started.

Some of the sentences could use restructuring or editing. E.g. lines 37-48 on p3 are full of clauses and commas that interfere with the flow.

p15, line 13 - taget -> target

Please provide an archival DOI for the version of STRONG used in the paper, via e.g. Zenodo.

**Reviewer 2**

Currently most metagenome assemblers or binners do not have specific functions for strain resolution, and as a result metagenome-assembled-genomes (MAGs) are often a mixture of genomes from similar strains. To overcome this limitation, Quince et al. developed a new algorithm, STRONG, that exploits Single-Copy Core Genes (SCGs) on the assembly graph to simultaneously solve the genomic sequences and abundance of different strains within a MAG. They tested the accuracy of STRONG by using both synthetic datasets and a realworld dataset and found supporting evidence that it is highly accurate. The manuscript was clearly written with sufficient technical details to follow. Compared with a previous solution developed by this group, STRONG demonstrated a significant improvement in accuracy.

Overall I think this work represents a significant amount of efforts to tackle such a difficult problem. I have a few suggestions that may further improve the work.

1. Synthetic datasets. Acknowledging that is impossible to design comprehensive synthetic datasets that reflect the diverse characteristics of a typical metagenome dataset, here it will be beneficial to include a few more critical ones. First, as the anaerobic digestor time series dataset indicates, strain-level balance may be static rather than dynamic in many microbial communities, a control experiment should be included to access the accuracy of STRONG in this context. Second, up to 5 strains per species were simulated in the synthetic datasets. This may limit the ability to explore the level of strain diversity and the accuracy of predicting them. Third, as STRONG relies on SCGs for strain resolution, it might help to remove strains with identical SCGs from the synthetic dataset to more accurately estimate its performance, at least the "not found" numbers should improve.

2. If the MAGs are processed one by one in the BayesPaths step, then in theory this step can be implemented in parallel so that the computing performance of this bottleneck could be improved. In addition, I suggest the authors to replace Concoct with MetaBAT 2, as Concoct is not very computational efficient (77 hours for the AD dataset, second bottleneck).

3. There is an alternative way used by the research community to identify strains. Instead of co-assembly of all the samples together, each sample is independently assembled tp avoid MAGs with a mixture of different strains. This approach, in theory, should only work well in cases where different strains dominant different samples, with the trade-off that the coverage of the species is lower for effective assemblies. I wonder if the authors want to include comparisons to this approach with their synthetic datasets.

**Author response**

'Reviewer #1: This paper introduces new software called STRONG, which enables strain resolved metagenomics using uncollapsed assembly graphs. The question of how to extract strain information present in metagenomes is an important research area that is at the leading edge of microbiome studies; STRONG potentially represents a substantial advance in research capabilities.

The underlying idea is clear, if not easy to implement: STRONG looks at the abundances of paths in cDBG unitigs across samples, and"resolves" paths through single copy gene sequences that best match the abundances, thus deconcolving them to match to different strains.

The paper is well organized, and the introduction and discussion are expert and excellent. The methods as presented also are clear and high quality. However, we struggled to understand exactly what the output of STRONG was, and how it could be used. Some specific questions here:

* what information does STRONG require to do its work? It seems like good quality genome assemblies/MAGs could be critical inputs, but we couldn't tell for sure.'

The STRONG pipeline generates MAGs using CONCOCT or alternatively Metabat2 (see below), strains are resolved for single-copy core genes on those MAGs and in that sense the MAG collection is a critical input since strains can only be resolved for MAGs. The actual strain resolution BayesPaths, however, could be applied more generally, it would in theory be possible to map SCG contigs to reference genomes if MAGs were not successfully constructed or even single-genes used without any binning.

'* If good bins are required, are there concerns about providing this input, in terms of STRONG's output quality?'

During the strain resolution, there is an additional SCG filtering step, so even contaminated MAGs should be processible. These and the above are both useful points to clarify so we have added some discussion of this to the manuscript (see lines 439-464 of the Discussion).

'* what is the actual primary output of STRONG? the strain by sample abundance matrix? Is anything done with non-single-copy-gene sequences?'

'* does STRONG provide insight into accessory/non-core elements of strains?'

We have added an additional final paragraph to the section STRONG pipeline (lines 151-155) that clarifies these two points and exactly what output is produced by STRONG.

'* is STRONG limited to bacteria and archaea by the single-copy-gene paradigm used?'

It is limited to prokaryotes in its current form because the gene calling and subgraph extraction assumes simple prokaryotic genes. It may be adaptable to eukaryotes but it would require further work and testing, Lines 443 – 449 now address this point specifically.

'* does STRONG work only on time series, or it can be used on spatial series (at least in theory)?'

This is also a good question, the answer is yes but with some caveats, this was discussed very briefly in the text already but we have elaborated on this point (see Discussion lines 488 - 491).

'* It seems like STRONG deals specifically with coding sequences (p20, line 8) in normal codon encodings. If so, that approach/limitation should be mentioned early on, as it limits STRONG to coding regions etc.'

The graph extraction operates at the level of ORFs so is indeed restricted to coding regions although the actual extracted graphs tend to include the upstream promoters. We can deal with any stop codons not just normal encodings. We are clear that STRONG uses single-copy core genes very early in the paper (see line 111 of Introduction) so we do not feel this needs extra emphasis. Especially as the actual strain resolution BayesPaths is not in theory restricted to coding regions.

Other major issues/questions --

'It seems like having STRONG built on top of DESMAN (STRONG postprocesses DESMAN output, we think?) would affect the comparison to DESMAN - that is, it's good it improves upon DESMAN, but would that not be expected? Are there other tools to compare to in terms of outputs? What kind of ground truth can be used to validate STRONG, if any?'

This is not actually the case, STRONG does not post-process DESMAN output, and we have tried to clarify this in the text, see Lines 176-177. STRONG refers to the complete pipeline from reads, to contigs and strain resolved MAGs. The focal algorithm for haplotype inference in STRONG is BayesPaths, for comparison purposes we also run DESMAN but the BayesPaths algorithm is completely independent to DESMAN. It also uses independent input. To obtain the input for DESMAN, we perform read mapping onto contigs to obtain variant frequencies. To obtain the input for BayesPaths we extract variant graphs from coassemblies and then use read threading to get unitig coverages. The algorithms themselves are also independent, DESMAN uses a Bayesian multinomial model to link independent variants into strain haplotypes and abundances. BayesPaths uses a variational Bayesian path finding algorithm on the variant graphs to resolves strain abundances and haplotypes.

In our implementation of the STRONG pipeline we try to synchronise the input to the two algorithms as much as possible using the same contigs, MAGs, and single-copy core genes within MAGs to enable a fair comparison but as emphasised above no actual input data is shared and no output feeds from one to the other.

We did manage to find two additional tools published very recently, one after initial submission of STRONG, inStrain (Olm et al. Nature Biotech 2021) and one published last year, mixtureS (Xin et al. Bioinf. 2020). The latter, mixtureS is a read based EM algorithm operating on a single sample, it generates predictions of haplotype numbers in a sample and the haplotypes themselves. We ran mixtureS on the synthetic communities by aggregating the individual samples from the data set, that seemed most reasonable, since it ensures that the strains are present in the same total coverage for all algorithms. The performance of mixtureS was inferior to both DESMAN and STRONG which given that it is quite similar to DESMAN in fundamentals indicates the advantage of using multiple samples.

We also evaluated inStrain but we decided not to include those results: inStrain presents itself as a tool for "rigorous detection of identical microbial strains", but it does not output either strain number or strain haplotype explicitly. It produces "strain clusters" which correspond to groupings of samples in which consistent SNV profiles are observed. In our synthetic communities these did not correspond well at all to the actual strain number. For instance, in one case for a MAG with five strains in different proportions across ten samples, inStrain failed to cluster any samples together. The implicit assumptions in the inStrain approach, shared with many other algorithms, for example StrainPhlan, is that one strain dominates each sample, therefore the strain mixtures that we are testing are outside their scope. We have added some discussion of this point (see lines 416-422).

The synthetic communities obviously have a ground truth, for real data comparisons are restricted to long reads and we do include a comparison to Nanopore data for the AD data set.

'Figure 2 was somewhat confusing in its presentation at first. If there is a way to use layout constraints or define the layout seed to keep haplo.0, haplo.1, and haplo.2 in the same layout but with different colors, that would make the figure clearer.'

That is a good suggestion and we have now revised the figure using the same graph layout throughout to make the figure clearer.

'It is unclear what information Figure 6 contributes specifically. We had a number of suggestions but aren't sure what the main message is, so please feel free to disregard these specific suggestions –

* perhaps limit the diagram to major phyla? Firmmicutes, Bacteroides, etc?

* We're not sure what the various FALSE etc. colors represent and they are impossible to distinguish on the figure.'

We have reformatted this figure as a rectangular heatmap which hopefully makes it clearer. The colours are just indicating which MAGs change significantly in abundance with time (either up or down) and which have significant changes in strain proportion.

'p15, line 48, we're not sure what "complete metagenome de novo strain resolution will become a realistic possibility" means in terms of the approach presented here. What would be the specific outcome - completely strain-resolved MAGs with haplotype linkages? Please define!'

We clarify this in the text as ‘obtaining the complete genomes of all significant clonal subpopulations within a species’ (Lines 517-519).

'The acknowledgements mention a contribution that seems significant enough for authorship.'

We respectfully disagree it was simply a short discussion and a paper utilising the method that inspired our graph extraction procedure is now in press, so we additionally cite that rather than adding an extra author.

Minor comments --

'Paragraph beginning at line 49, p2 - it seems like strain variation belongs in here as a third reason? Also, after citation [17], a new sentence could be started.'

'Some of the sentences could use restructuring or editing. E.g. lines 37-48 on p3 are full of clauses and commas that interfere with the flow.'

We have tried to improve the flow of the text here.

'p15, line 13 - taget -> target'

Fixed.

'Please provide an archival DOI for the version of STRONG used in the paper,'

via e.g. Zenodo.

We have created an archival version <http://doi.org/10.5281/zenodo.4740331>

'Reviewer #2: Currently most metagenome assemblers or binners do not have specific functions for strain resolution, and as a result metagenome-assembled-genomes (MAGs) are often a mixture of genomes from similar strains. To overcome this limitation, Quince et al. developed a new algorithm, STRONG, that exploits Single- Copy Core Genes (SCGs) on the assembly graph to simultaneously solve the genomic sequences and abundance of different strains within a MAG. They tested the accuracy of STRONG by using both synthetic datasets and a realworld dataset and found supporting evidence that it is highly accurate. The manuscript was clearly written with sufficient technical details to follow. Compared with a previous solution developed by this group, STRONG demonstrated a significant improvement in accuracy.'

'Overall I think this work represents a significant amount of efforts to tackle such a difficult problem. I have a few suggestions that may further improve the work.'

'1. Synthetic datasets. Acknowledging that is impossible to design comprehensive synthetic datasets that reflect the diverse characteristics of a typical metagenome dataset, here it will be beneficial to include a few more critical ones. First, as the anaerobic digestor time series dataset indicates, strain-level balance may be static rather than dynamic in many microbial communities, a control experiment should be included to access the accuracy of STRONG in this context. Second, up to 5 strains per species were simulated in the synthetic datasets. This may limit the ability to explore the level of strain diversity and the accuracy of predicting them. Third, as STRONG relies on SCGs for strain resolution, it might help to remove strains with identical SCGs from the synthetic dataset to more accurately estimate its performance, at least the "not found" numbers should improve.'

We will respond to each of the points raised by the reviewer in turn. We agree stable strain proportions do appear to be a real phenomenon, so we added three new synthetic data sets where we use a hierarchical data set generation procedure to investigate increasing levels of strain stability (see new section lines 270 to 298).

STRONG can still get approximately the correct number of strains, and good predictions for the stable strain proportions but the sequence error rate increases. We have added discussion of this to the paper. Secondly, we have added four new data sets with higher strain numbers up to ten strains in total (see lines 243 – 269).

STRONG performs quite well at estimating strain numbers in these more complex data sets, and for some MAGs obtains up to eight or nine strains that match to the references but again the average error rate is increased although it was still only 0.18% for the ten sample data set. Thirdly we do filter identical strains on SCGs as part of the strain selection procedure, with a minimum ANI divergence of 0.05% although that is quite low (5 in 10,000 bps) and after SCG filtering as discussed in the text some strains are identical. However, it is not clear to us whether these should be considered divergent or not. They were prior to filtering so we still count them as ‘Not Found’.

'2. If the MAGs are processed one by one in the BayesPaths step, then in theory this step can be implemented in parallel so that the computing performance of this bottleneck could be improved. In addition, I suggest the authors to replace Concoct with MetaBAT 2, as Concoct is not very computational efficient (77 hours for the AD dataset, second bottleneck).'

The STRONG pipeline runs each MAG independently so that is indeed automatically parallelised as part of the SnakeMake pipeline. We have done as the reviewer suggested and added Metabat2 as an alternative binner to Concoct which we prefer to keep as the default option since at least in our experience on real data it gives better results.

'3. There is an alternative way used by the research community to identify strains. Instead of co-assembly of all the samples together, each sample is independently assembled tp avoid MAGs with a mixture of different strains. This approach, in theory, should only work well in cases where different strains dominant different samples, with the trade-off that the coverage of the species is lower for effective assemblies. I wonder if the authors want to include comparisons to this approach with their synthetic datasets.'

As discussed above we did consider adding a comparison to inStrain which works on this principle but any such method that resolves only the dominant strain will be of limited effectiveness on precisely the type of data sets we investigate here i.e. where mixtures are present and some strains are not dominant anywhere. We could have devised a data set where such an approach would work but given that in real data you will not know if subdominant strains are missed it seemed to us to not be a worthwhile exercise. Furthermore, even having resolved strains in this way, you would still need an additional step to estimate their abundances across samples, which itself is not trivial. On the whole we felt it was better not to include this comparison

**2nd round**

**Reviewer 1**

. I appreciate the significant revisions and have no further comments!

Note that I myself was not sure that there was any way to compare to "ground truth" (it's a problem we have in this area as well) and I am quite happy with the efforts made by the authors.

**Reviewer 2**

The revised version has sufficiently addressed my previous critiques.
